# Altered distribution of intervertebral disc degeneration in degenerative thoracolumbar kyphosis: A retrospective clinical study and finite element analysis

**DOI:** 10.1097/MD.0000000000049770

**Published:** 2026-07-10

**Authors:** Ruifeng Xun, Kangkang Wang, Wanmei Yang, Ao Ding, Jian Wang, Ji Li, Wei Zhang, Xilong Cui, Haiyang Yu

**Affiliations:** a Department of Orthopedics, Fuyang People’s Hospital Affiliated to Anhui Medical University, Fuyang, Anhui, China; b Department of Orthopaedics, Linquan County People’s Hospital, Fuyang, Anhui, China; c Department of Orthopedics, Chaohu Hospital Affiliated to Anhui Medical University, Chaohu, Anhui, China; d Department of Orthopedics, The First Clinical College, Guangdong Medical University, Zhanjiang, Guangdong, China; e Department of Clinical Medical College, Anhui Medical University, Hefei, Anhui, China.

**Keywords:** finite element analysis, global kyphosis, intervertebral disc pressure, thoracolumbar kyphosis

## Abstract

This study aimed to systematically characterize the unique topographic pattern of disc degeneration in degenerative thoracolumbar kyphosis (DTLK) and to elucidate its underlying biomechanical basis using combined clinical and finite element analyses. This is a retrospective clinical study combined with finite element analysis (FEA). This clinical study included 170 DTLK patients and 153 controls for analysis. Three finite element models were constructed for biomechanical simulation. We compared the 2 groups for both the grade of intervertebral disc degeneration and the distribution of degenerated segments across the T11–S1 levels. Finite element models of the T10–S1 segment were constructed for the normal, DTLK, and global kyphosis (GK) groups. The intervertebral disc pressure (IDP) from T11 to S1 were analyzed under standing, flexion, extension, lateral bending, and axial rotation. Regarding the overall degeneration grade, Grade IV and V degenerations were both significantly more prevalent in DTLK than in controls (Z = −2.232, *P* = .026 and Z = 4.504, *P* < .01, respectively), with grade V showing a more pronounced group difference. Grade IV and V disc degenerations occurred predominantly at the L1–L2 (*P* < .001), L2–L3 (*P* < .001), L3–L4 (*P* < .001), and L4–L5 (*P* < .001) segments in the DTLK group. FEA revealed that in both the DTLK and GK models, the IDP from T11 to L5 exceeded that of the normal model under standing, flexion, extension, lateral bending, and rotation conditions. The IDP at the apical L2–L3 segment in normal models were 0.21, 0.34, 0.15, 0.32, 0.32, 0.30, and 0.29 MPa during standing, flexion, extension, left bending, right bending, left rotation and right rotation. In the DTLK model, the corresponding values are 0.33, 0.45, 0.25, 0.42, 0.41, 0.39, and 0.40 MPa. In contrast, in the GK model, the IDP are 0.58, 0.76, 0.42, 0.64, 0.64, 0.63, and 0.61 MPa. DTLK exhibits a distinctive pattern of intervertebral disc degeneration predominantly localized to the L1–L4 segments, contrasting sharply with the L4–S1 predominance observed in normal aging. FEA indicates that the high-stress zone of the DTLK disc is located in the thoracolumbar kyphotic segment.

## 1. Introduction

Degenerative thoracolumbar kyphosis (DTLK) is one of the common subtypes of spinal deformity in adults and seriously affects the quality of life. Its pathological process involves intervertebral disc degeneration (IDD), vertebral wedge deformation and sagittal plane imbalance.^[1,2]^ If the DTLK progresses further, it can develop into global kyphosis (GK) with more significant pain, dysfunction and nerve damage.^[3]^ IDD plays a key role in this process, which is not only a sign of structural degeneration, but also a direct reflection of mechanical environment changes.^[4]^ Previous studies have focused on the degeneration patterns of intervertebral discs in scoliosis or post-traumatic deformities, but systematic studies on the distribution characteristics and mechanical mechanisms of IDD in DTLK, a specific type of deformity, are still lacking.^[5]^

There is a lack of clinical big data analysis on the distribution patterns of IDD segments in DTLK patients, as well as in-depth research to explain its causes from the biomechanical perspective. Biomechanical parameters, particularly intervertebral disc pressure (IDP) and von Mises stress, serve as predictive indicators of the severity of IDD. Finite element analysis (FEA) is the primary method for quantifying these parameters.^[6]^ Zhang Y et al demonstrated that FEA can effectively assess the degree of IDD by elucidating the role of the cartilage endplate in its quasi-static biomechanical behavior.^[7]^ Somtua C et al employed the finite element method to analyze the stress distribution pattern of scoliosis within the 30° to 70° range of the Cobb angle, with the maximum equivalent total strain occurring at the center of spinal curvature.^[8]^ The 2022 study by Matsumoto K et al demonstrated that sagittal plane imbalance increases disc stress and the risk of compression fractures, highlighting the critical importance of sagittal alignment in spinal biomechanics.^[9]^ However, these findings specific to scoliosis cannot be directly applied to kyphosis. This study fills this research gap by establishing specialized DTLK models and models of their progression to GK.

This study aimed to compare the distribution of IDD levels between DTLK patients and normal population through retrospective analysis. Three spinal models, normal, DTLK and GK, were constructed by finite element method to simulate the pressure and stress distribution in intervertebral discs under standing and various physiological loads, so as to explore and provide a biomechanical explanation for the distinct disc degeneration pattern observed in DTLK. The research methods include clinical image data analysis and 3-dimensional finite element modeling and verification, in order to comprehensively explain the degeneration characteristics of DTLK from the 2 dimensions of structure and mechanics.

## 2. Methods

### 2.1. Clinical study of IDD distribution characteristics in patients with DTLK

#### 2.1.1. Patient recruitment and inclusion/exclusion criteria

The imaging data used in this study was obtained on August 15, 2025. This study was approved by the Ethics Committee of Fuyang People’s Hospital before implementation (NO. 2024-223). The individual participant information could be identified during or after the data collection process. A retrospective analysis was performed on the IDD grade of 170 patients diagnosed as DTLK in Fuyang People’s Hospital from April 2017 to June 2025 and 153 people in physical examination. The grading of disc degeneration was determined by the Pfirrmann classification.^[10]^ Two experienced spinal surgeons independently assessed the magnetic resonance imaging (MRI) images and graded the discs from T11 to S1 for the DTLK group and control group (CG). Any discrepancies were resolved through discussion or by consultation with a third senior expert. The inter-rater reliability was evaluated using the intraclass correlation coefficient, which showed excellent agreement (intraclass correlation coefficient = 0.91).

The inclusion criteria of DTLK group included: confirmed as DTLK by whole spine X-ray examination; Cobb angle of kyphosis >15 at T10-L2 segment; and complete and clear preoperative standing full-length anteroposterior X-ray and MRI of lumbar.^[11]^ All patients underwent comprehensive clinical evaluation, including physical examination for sagittal imbalance symptoms and radiological confirmation. The diagnosis was established through a combination of clinical manifestations and radiological findings, rather than solely based on imaging alone. Exclusion criteria are as follows: incomplete MRI or X-ray imaging data; diagnosis of idiopathic scoliosis, congenital scoliosis or degenerative scoliosis with kyphosis deformity; presence of spinal fracture, tumor or infection; and history of spinal surgery. The CG consisted of people who underwent lumbar MRI and full-length anteroposterior spine films during physical examination in our hospital. These individuals underwent imaging examinations to evaluate nonspecific low back pain or as part of routine health screening, and were confirmed by 2 senior spinal surgery specialists to have no structural spinal deformities. The inclusion criteria were as follows: no structural spinal deformity was found by whole spine X-ray examination; Cobb angle of kyphosis in T10-L2 segment was between 0 and 15; and complete and clear preoperative standing full-length anteroposterior and lateral X-ray films and lumbar MRI images. The exclusion criteria are as follows: incomplete MRI or X-ray imaging data; diagnosed as idiopathic scoliosis, congenital scoliosis or degenerative scoliosis with kyphosis deformity; presence of spinal fracture, tumor or infection; and history of spinal surgery.

### 2.2. Finite element analysis

#### 2.2.1. Establishment of normal finite element models

**Figure 1.**
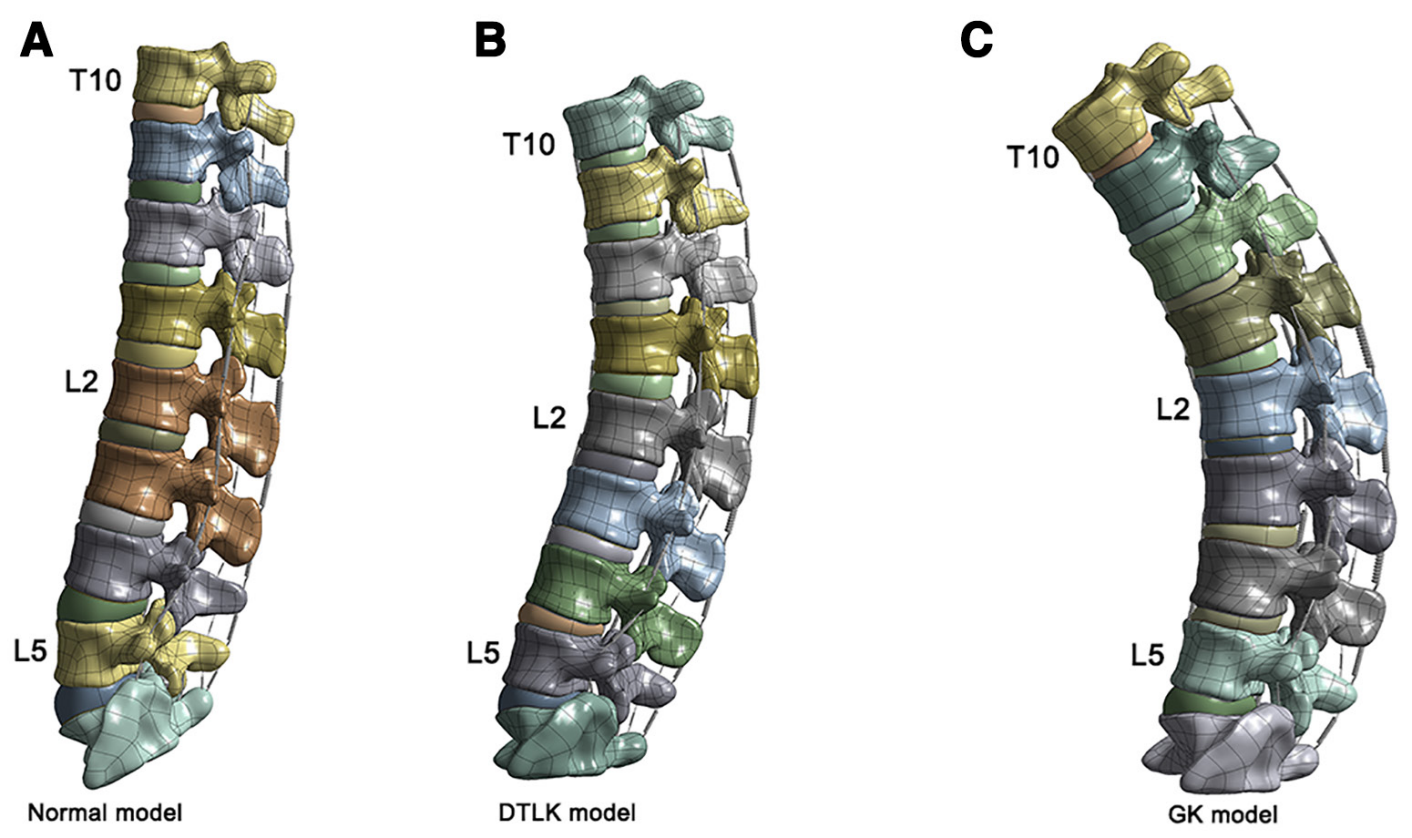
Finite element models developed using different software platforms. (A) Normal model. (B) DTLK model. (C) GK model. The model colors are used solely to illustrate the anatomical structure and do not represent stress magnitude, biomechanical parameters, or model-specific attributes. DTLK = degenerative thoracolumbar kyphosis, GK = global kyphosis.

The normal finite element model (Fig. 1) was constructed from CT data of a healthy 32-year-old female volunteer (height 166 cm, weight 53 kg, BMI 19.2 kg/m^2^). The volunteer exhibited a thoracolumbar kyphotic Cobb angle (T12–L2) of 9° and a lumbar lordotic Cobb angle (L1–S1) of 30°, with no structural spinal deformity. CT images were acquired at 0.625 mm slice thickness, covering the T10–S1 region. The Digital Imaging and Communications in Medicine-formatted CT image data were first imported into the 3-dimensional reconstruction software Mimics 21.0 to generate multi-slice continuous images in the coronal, sagittal, and axial planes. An appropriate grayscale threshold was applied to enhance the visibility of bony structures. Next, the T10–S1 vertebral segments were segmented based on grayscale thresholds to construct a preliminary 3-dimensional geometric model. The 3-dimensional model was then smoothed using Geomagic Studio 2017 software and converted into an accurate NURBS surface model representing the anatomical surfaces. Finally, the model was exported in STEP format and imported into SolidWorks 2024 for further processing and assembly. The cortical bone and endplate were modeled with a thickness of 0.5 mm. The intervertebral disc is composed of the nucleus pulposus and the annulus fibrosus. The nucleus pulposus is located internally, while the annulus fibrosus is situated externally. The nucleus pulposus accounts for approximately 40% of the total volume of the intervertebral disc and is positioned centrally and posteriorly within the annulus fibrosus. The methods for manufacturing the nucleus pulposus and the annulus fibrosus were accomplished in SolidWorks.^[12,13]^ The facet joints were modeled with an articular cartilage thickness of 0.5 mm and an initial joint gap of 0.5 mm. A fixed contact relationship was defined between the vertebral body, endplate, and intervertebral disc. The contact between articular cartilages was modeled as frictionless.

### 2.3. Modeling of disc degeneration

To reflect the material changes associated with disc degeneration in the DTLK and GK models, the material properties of the nucleus pulposus and annulus fibrosus at specific segments were adjusted according to Table 1. Clinical observations revealed that the highest proportion of Grade V degeneration was observed in the L1–L2 and L2–L3 segments of DTLK patients (56.4% and 58.8%, respectively). We employed a hierarchical progressive modeling strategy to simulate the biomechanical characteristics of DTLK at different stages: the normal model represents the healthy state; the DTLK model simulates the transitional phase during degenerative progression. Given that patients with DTLK often present with multi-segmental spinal degeneration, the DTLK model adopts a “moderate degeneration” criterion across all segments. The GK model simulates the terminal stage of deformity, assigning the L1–L2 and L2–L3 segments to “severe degeneration” while the remaining segments are classified as “moderate degeneration.” This stepwise grading design systematically captures the progressive biomechanical deterioration from a healthy state through mild deformity to severe deformity.

**Table 1:** Material properties of the different structures used for the FE model.

Components	Young modulus (MPa)	Poisson ratio	Linear stiffness (N/mm)
Vertebra Cortical bone	12,000	0.3	/
Cancellous bone	100	0.3	/
Endplate	25	0.25	/
Cartilage	50	0.3	/
Nucleus pulposus	1	0.499	/
Annulus fibrosis	4.2	0.453	/
Moderate degeneration of nucleus pulposus	1.26	0.45	/
Severe degeneration of nucleus pulposus	1.66	0.4	/
Moderate degeneration of annulus fibrosis	5	0.4	/
Severe degeneration of annulus fibrosis	12.29	0.35	/
Anterior longitudinal ligament	/	/	8.74
Posterior longitudinal ligament	/	/	5.83
Interspinous ligament	/	/	10.85
Ligament flavum	/	/	15.98
Intertransverse ligament	/	/	0.19
Supraspinous ligament	/	/	3.82

### 2.4. Ligament

The anterior longitudinal ligament, posterior longitudinal ligament, ligamentum flavum, supraspinous ligament, interspinous ligament, and intertransverse ligament were simulated using linear springs subject only to pullout force.

### 2.5. Material properties

Finite element preprocessing tasks, including material property assignment, ligament modeling, mesh generation, contact relationship definition, and application of load and boundary conditions, were performed using ANSYS software. The material properties of each tissue were assigned based on previously published data, as summarized in Table 1.^[14–17]^

### 2.6. Mesh convergence and model deformation

Mesh convergence analysis was performed to ensure solution independence from discretization. The convergence criterion was defined as < 5% change in von Mises stress and maximum IDP at the L2–L3 segment (the apical region) between successive mesh refinements. Starting from an initial element size of 3.0 mm, 3 iterations of adaptive mesh refinement were conducted, reducing element size by 30% at each step. The normal models consist of 1216,325 nodes and 755,358 elements. The cortical bone convergence was achieved at an element size of 1.5 mm for cortical bone and 1.0 mm for intervertebral discs, with final model comprises 527,248 nodes and 317,513 elements, while the cancellous bone consists of 348,214 nodes and 238,205 elements. Following mesh convergence analysis, a total of 1216,325 nodes and 755,358 elements were included. Using 3-matic software, the DTLK and GK models were generated by adjusting the intervertebral disc height and sagittal alignment of each vertebral body. The DTLK and GK models were constructed by systematically reducing the intervertebral disc height and adjusting the sagittal alignment in the 3-matic software. Through reducing the disc height and modifying the sagittal alignment of the vertebrae, the DTLK finite element model with a cobb angle of 30° and the GK finite element model with a cobb angle of 40° were ultimately obtained. The normal, DTLK, and GK models, along with their detailed components, are illustrated in Figure 1. The DTLK model comprises 1176,955 nodes and 729,712 elements: cortical bone accounts for 529,416 nodes and 319,483 elements, and trabecular bone for 351,953 nodes and 241,338 elements. The GK model comprises 1151,383 nodes and 711,439 elements: cortical bone accounts for 536,734 nodes and 323,886 elements, and cancellous bone for 350,624 nodes and 240,218 elements.

### 2.7. Boundary and loading conditions

The lower surface of the S1 vertebral body was selected as the fixation point. According to the loading and boundary conditions reported in the literature,^[18]^ a torque of 7.5 Nm and an axial compressive force of 500 N, applied perpendicular to the *z*-axis, were imposed on the upper endplate surface of T10 to simulate the flexion, extension, lateral bending and rotation activities of spinal motion. The stress distribution under the flexion, extension, lateral bending and rotation activities of the finite element model was analyzed.

### 2.8. Measurements and assessment indices

The average intradiscal pressure and maximum von Mises stress data were collected and analyzed for the 6 activities of the spine.

### 2.9. Statistical analysis

Descriptive statistics for continuous data were presented as mean ± standard deviation ($\bar{x}\pm s$). Categorical data in this study were expressed as percentages. Normality of continuous variables (age and BMI) was assessed using the Shapiro–Wilk test. Between-group comparisons for normally distributed continuous variables were performed using independent samples *t*-tests, while Mann–Whitney *U* tests were used for non-normally distributed data. Categorical variables (sex distribution) were compared using Chi-square tests. For IDD grade comparisons between groups, the Mann–Whitney *U* test was applied due to the ordinal nature of the Pfirrmann grading system. The level of significance was set at *P* < .05.

## 3. Results

### 3.1. General information

As shown in Table 2, according to the preset inclusion criteria, a total of 170 patients with degenerative kyphosis were included in this study, including 49 males and 121 females, with an age range of 55 to 87 (mean, 68.48 ± 6.60) years. The CG consisted of 153 individuals undergoing physical examinations, including 38 males and 115 females aged 53 to 79 (mean, 66.14 ± 6.13) years. There were no statistically significant differences observed in age, gender, and BMI between the DTLK group and CG (*P* > .05).

**Table 2:** Comparison of age, gender, and BMI parameters between the DTLK group and the control group.

Group	Sex		BMI (kg/m^2^)	Age (years)
	Male (n)	Female (n)		
DTLK group	49	121	24.56 ± 3.23	68.48 ± 6.60
Control group	38	115	23.96 ± 3.15	66.14 ± 6.13
*P*	>.05		>.05	>.05

DTLK = degenerative thoracolumbar kyphosis.

### 3.2. IDD grades

**Figure 2.**
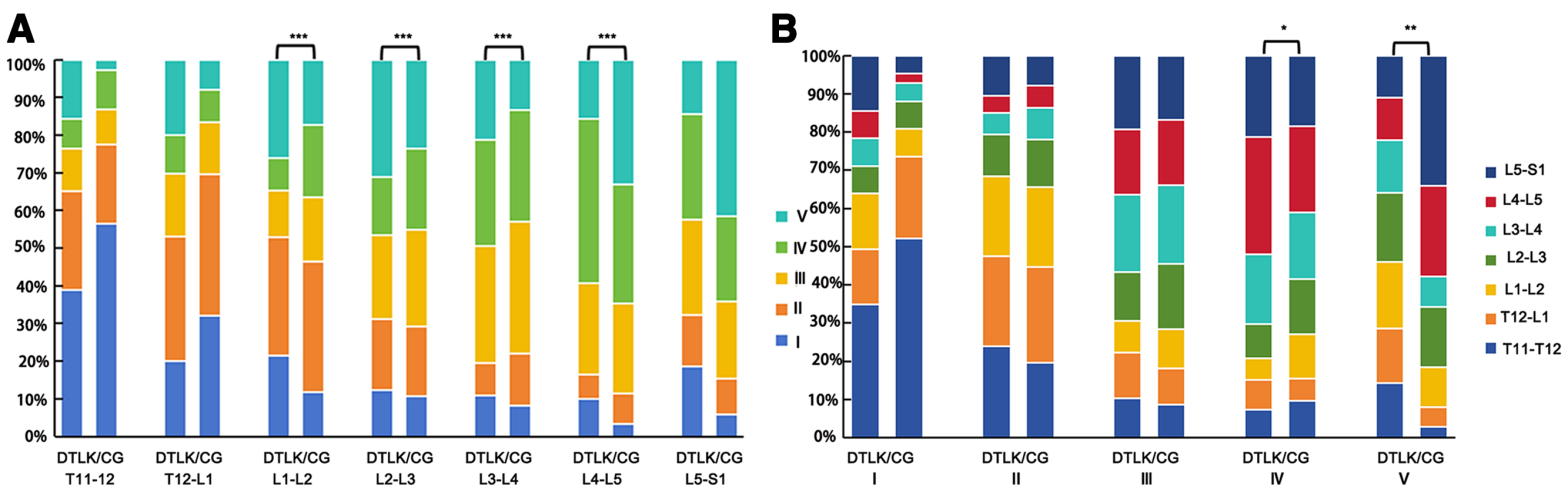
Intervertebral disc degeneration (IDD) grade distribution in the T11–S1 segment. (A) Overall distribution of Pfirrmann grades (I–V) in the DTLK and control groups, expressed as percentages of total discs assessed. (B) Segmental distribution of IDD grades across T11–S1 levels in each group. Asterisks indicate statistical significance: *P < .05, **P < .01, ***P < .001. CG = control group, DTLK = degenerative thoracolumbar kyphosis.

A total of 1190 intervertebral discs from 170 DTLK patients and 1071 discs from 153 control subjects were graded. As shown in Figure 2A, the DTLK group exhibited a markedly skewed distribution toward severe degeneration: grade IV–V accounted for 62.4% (743/1190) of all discs, whereas in the CG, grade IV–V represented only 30.8% (330/1071). Conversely, mild-to-moderate degeneration (grades I–III) comprised 37.6% of discs in the DTLK group versus 69.2% in the CG.

The site-specific distribution of degeneration grades is presented in Table 3 and Figure 2B. In the DTLK group, severe degeneration (grades IV–V) was predominantly localized to the L1–L2 (107/170, 62.9%), L2–L3 (117/170, 68.8%), L3–L4 (111/170, 65.3%), and L4–L5 (120/170, 70.6%) segments. By contrast, in the CG, grades IV–V were most prevalent at L4–L5 (75/153, 49.0%) and L5–S1 (67/153, 43.8%). The DTLK group showed significantly higher rates of severe degeneration at L1–L2, L2–L3, L3–L4, and L4–L5 compared with controls.

**Table 3:** IDD grade and distribution in the degenerative thoracolumbar kyphosis and control groups.

	Total	Segment							Statistical results	
		T_11_–T_12_	T_12_–L_1_	L_1–_L_2_	L_2_–L_3_	L_3_–L_4_	L_4_–L_5_	L_5_–S_1_	Z value	*P*
I										
DTLK	14 (1.2)	5 (2.9)	2 (1.1)	2 (1.1)	1 (0.5)	1 (0.5)	1 (0.5)	2 (1.1)	−1.494	.135
CG	42 (3.9)	22 (14.3)	9 (5.8)	3 (1.9)	3 (1.9)	2 (1.3)	1 (0.6)	2 (1.3)		
II										
DTLK	200 (16.8)	48 (28.2)	47 (27.6)	42 (24.7)	22 (12.9)	11 (6.4)	9 (5.2)	21 (12.3)	0.806	.420
CG	378 (35.3)	74 (48.3)	95 (62)	79 (51.6)	47 (30.7)	31 (20.2)	22 (14.3)	30 (19.6)		
III										
DTLK	233 (19.6)	24 (14.1)	28 (16.4)	19 (11.1)	30 (17.6)	47 (27.6)	40 (23.5)	45 (26.4)	−0.184	.854
CG	321 (30.0)	28 (18.3)	30 (19.6)	33 (21.5)	55 (35.9)	66 (43.1)	55 (35.9)	54 (35.2)		
IV										
DTLK	192 (16.1)	14 (8.2)	15 (8.8)	11 (6.4)	17 (10)	35 (20.5)	59 (34.7)	41 (24.1)	−2.232	.026
CG	292 (27.3)	28 (18.3)	17 (11.1)	34 (22.2)	42 (27.4)	51 (33.3)	66 (43.1)	54 (35.2)		
V										
DTLK	551 (46.3)	79 (46.4)	78 (45.8)	96 (56.4)	100 (58.8)	76 (44.7)	61 (35.8)	61 (35.8)	4.504	<.01
CG	38 (3.5)	1 (0.6)	2 (1.3)	4 (2.6)	6 (3.9)	3 (1.9)	9 (5.8)	13 (8.4)		
*Z* value	/	−2.071	−1.820	−8.255	−8.716	−7.660	−5.933	−4.428	/	/
*P*	/	.413	.286	<.001	<.001	<.001	<.001	.374	/	/

Data are presented as n (%), where n is the number of patients with the indicated grade at each segment, and % is the proportion within each group (DTLK: n = 170; CG: n = 153). Overall grade distributions in the Results are based on total disc numbers (DTLK: 1190; CG: 1071). Total grade percentages are calculated as (number of discs at each grade/ total number of discs in group) × 100%. Between-group comparison for each segment: Mann–Whitney *U* test.

CG = control group, DTLK = degenerative thoracolumbar kyphosis, IDD = intervertebral disc degeneration.

### 3.3. FE model validation

**Figure 3.**
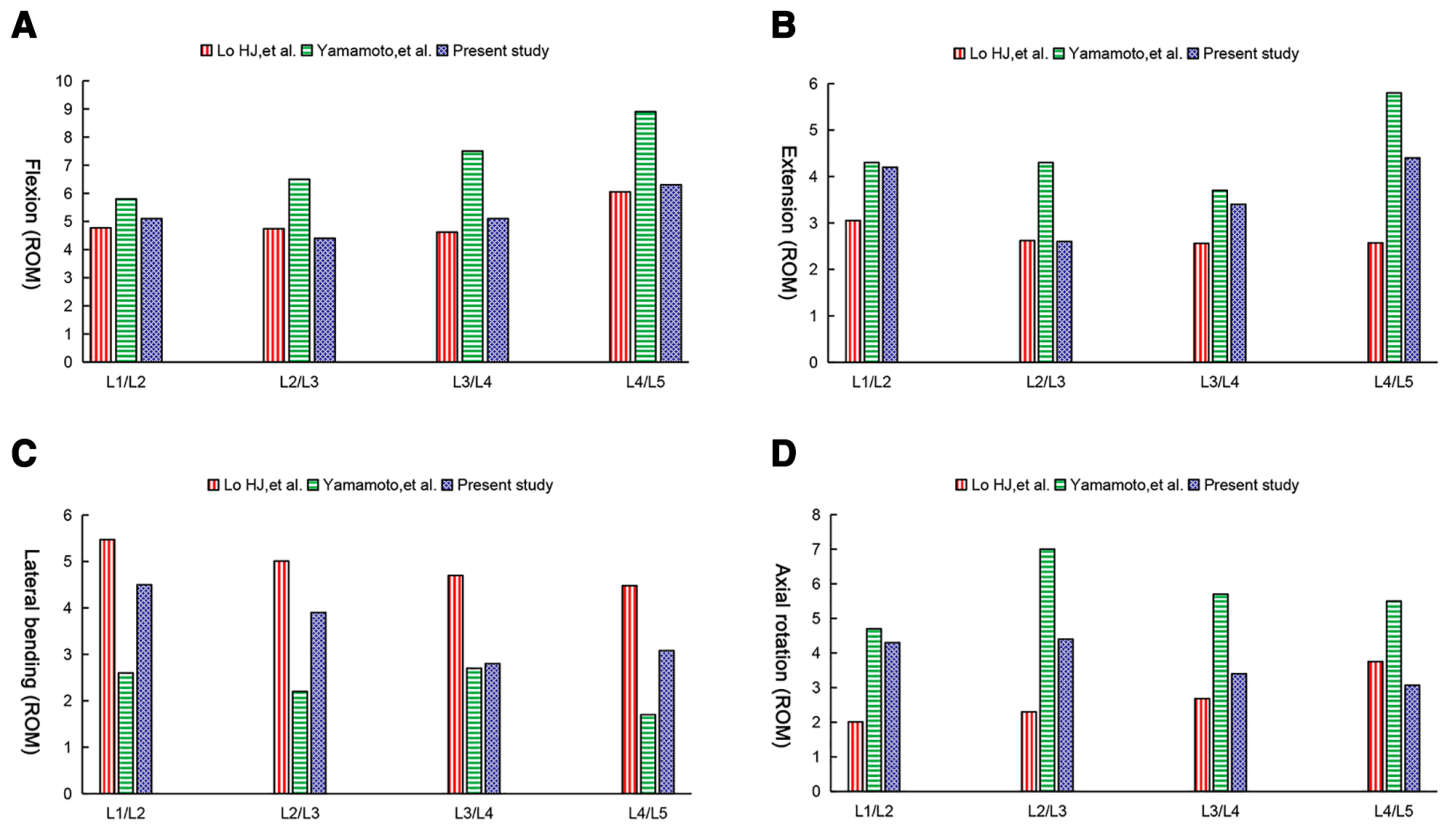
Comparison of our intervertebral ROM of the normal finite element model with previous studies. (A) Flexion, (B) Extension, (C) Lateral bending, (D) Axial rotation. ROM = range of motion.

**Figure 4.**
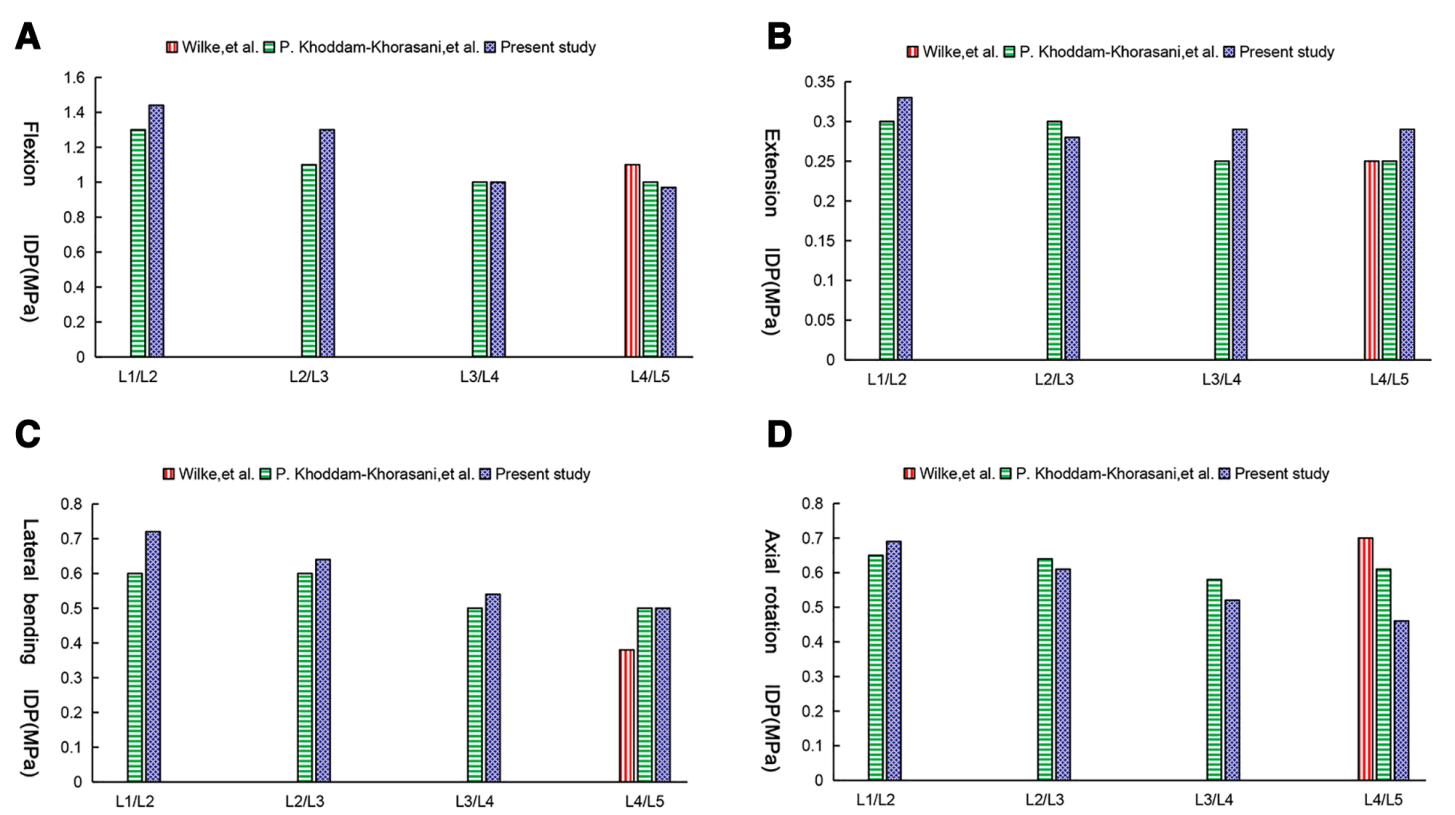
Comparison of our IDP of the normal finite element model with previous studies. (A) Flexion, (B) Extension, (C) Lateral bending, (D) Axial rotation. IDP = intervertebral disc pressure.

A force and torque of 150 N vertically down with a torque of 10 Nm was applied to the upper endplate of the L1 vertebral body. The range of motion of each lumbar segment was obtained under this condition. The results were compared with those of Yamamoto and Lo HJ et al.^[19,20]^ The results showed that the motion range of each segment of the lumbar spine in the normal model under different physiological activity states was basically consistent with the conclusions of previous studies, as shown in Figure 3. The base of the L5 vertebral body was fixed, on the upper end plate of the L1 vertebra, the following forces were applied: a flexion force of 1175 N and a torque of 7.5 Nm; an extension force of 500 N and a torque of 7.5 Nm; a lateral bending force of 700 N and a torque of 7.8 Nm; and an axial rotation force of 700 N with a torque of 5.5 Nm. The stress levels of the 4 intervertebral discs in the L1–L5 segment were subsequently measured.^[21,22]^ As is shown in Figure 4, the results were compared with the results in the literature. The results are generally consistent with the literature results.

### 3.4. Average IDP of the T11–S1 segment in 3 FE models

**Figure 5.**
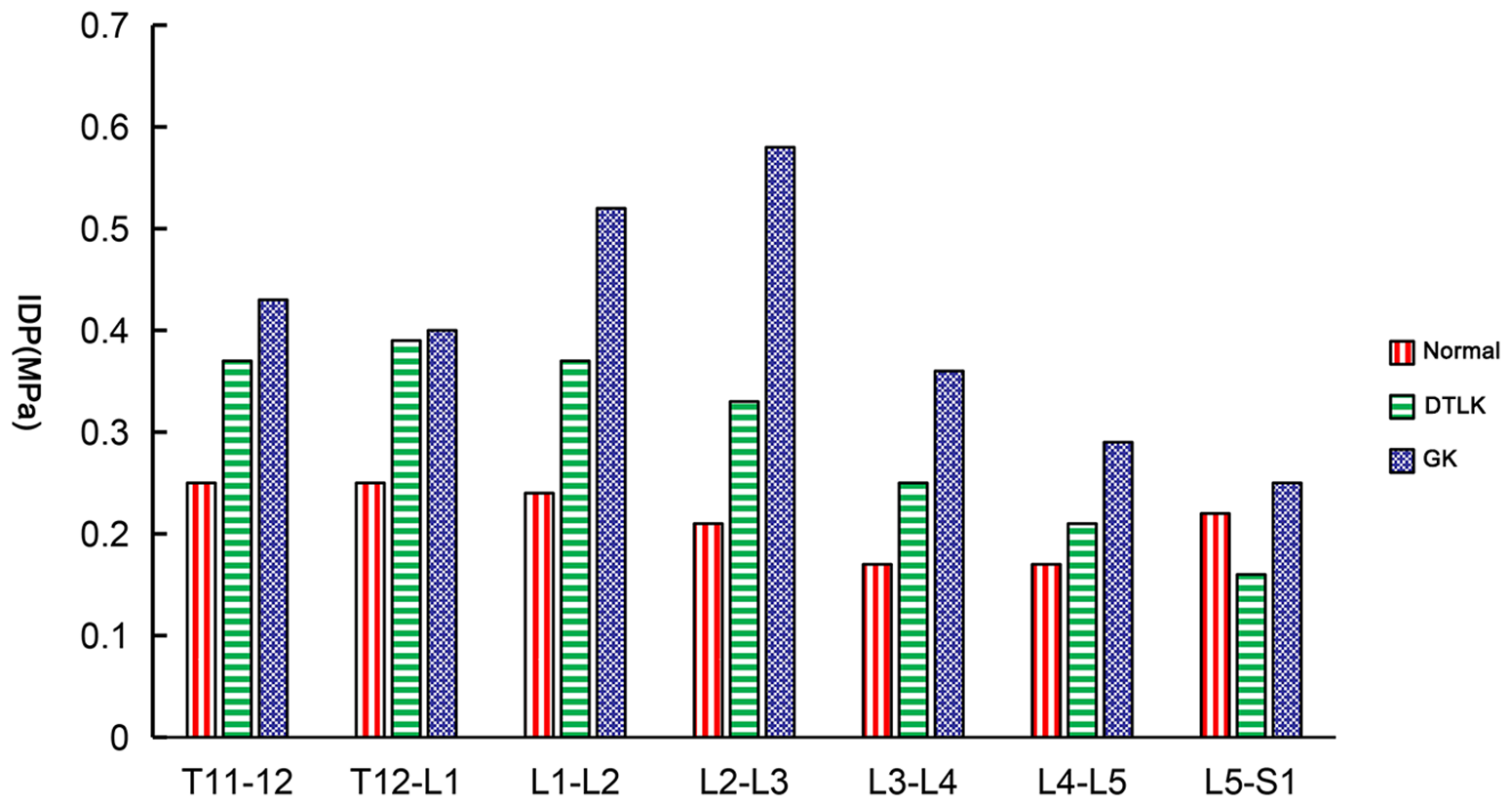
The intervertebral disc pressure (IDP) in the standing position across T11–S1 segments in the 3 finite element models. DTLK = degenerative thoracolumbar kyphosis, GK = global kyphosis.

**Figure 6.**
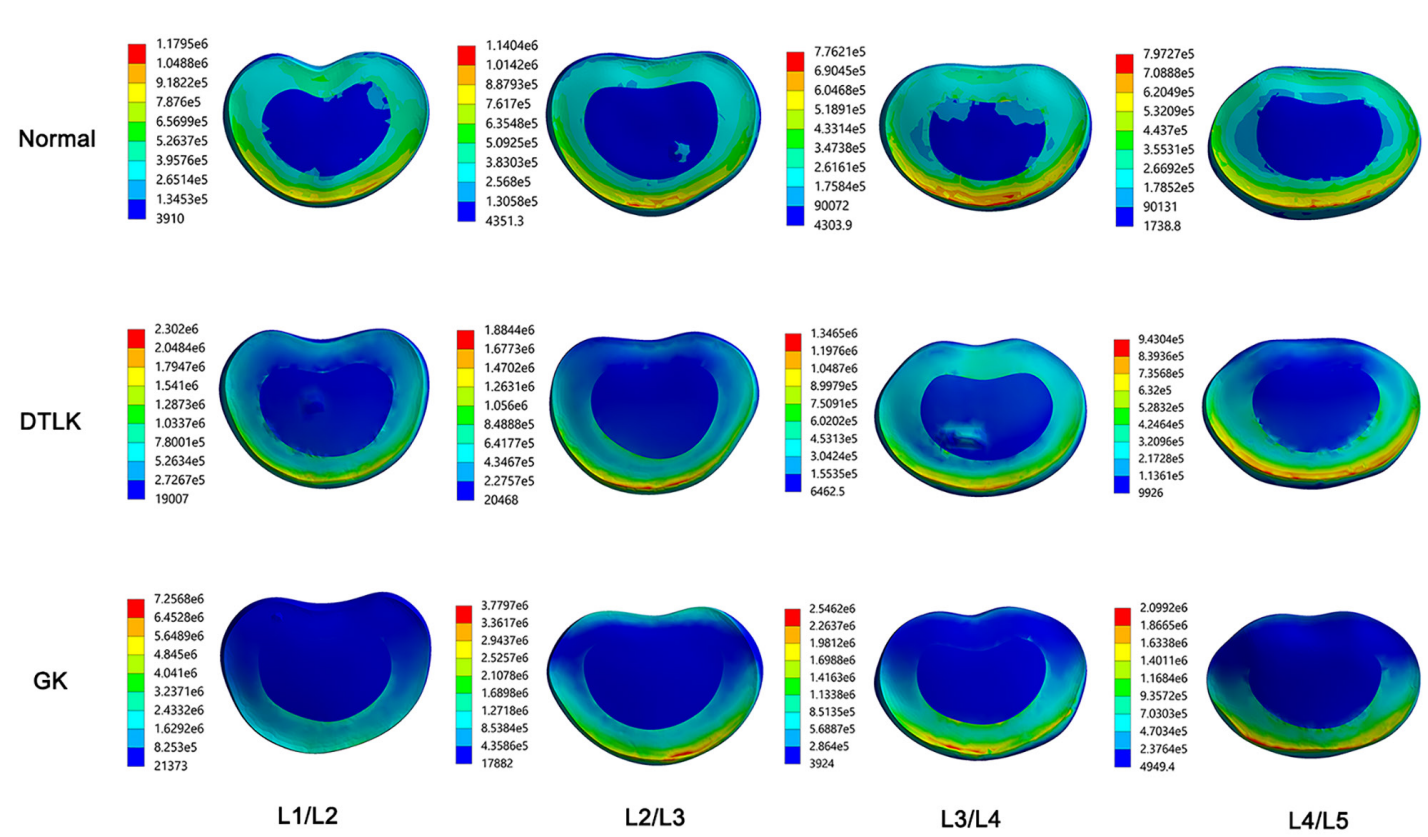
The stress distributions of L1–L4 segment in 3 finite element models in standing position. The stress values are presented in MPa. DTLK = degenerative thoracolumbar kyphosis, GK = global kyphosis.

**Figure 7.**
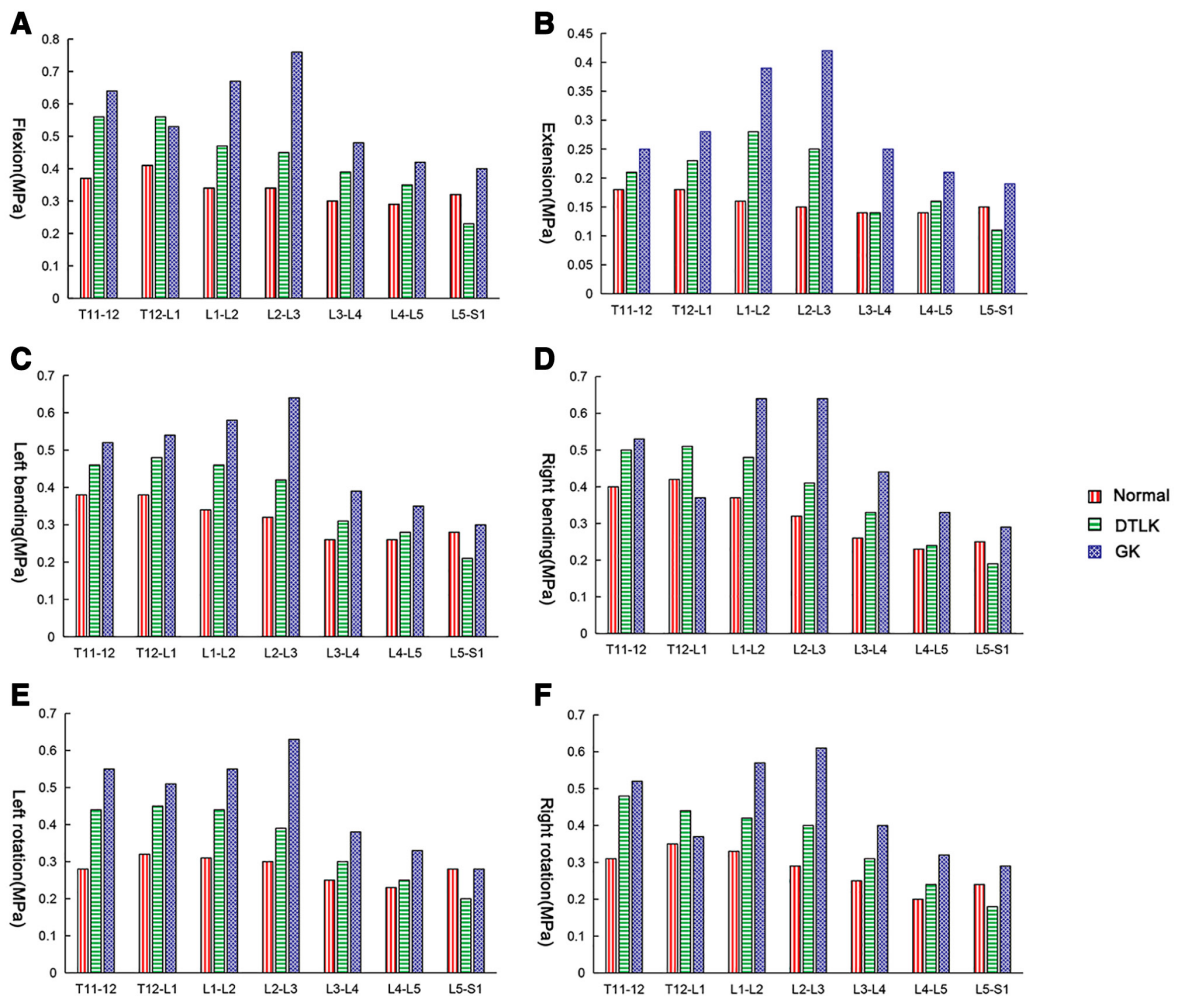
The IDP of the T11–S1 segment in the 3 finite element models under flexion, extension, lateral bending, and axial rotation. (A) Flexion, (B) Extension, (C) Left bending, (D) Right bending, (E) Left rotation, (F) Right rotation. DTLK = degenerative thoracolumbar kyphosis, GK = global kyphosis.

**Figure 8.**
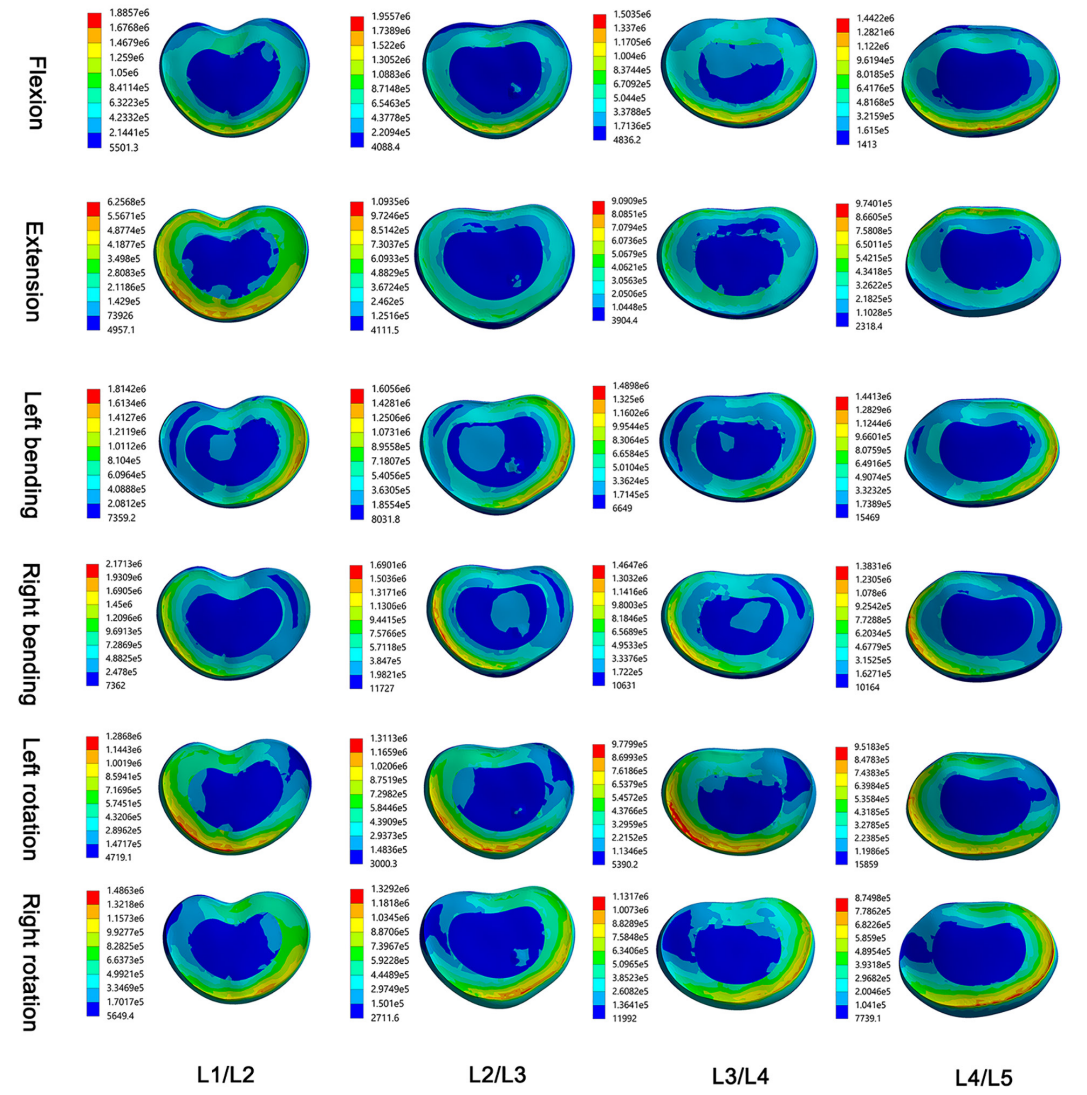
Stress distribution of intervertebral discs under flexion, extension, bending and rotation in the normal model. The stress values are presented in MPa.

**Figure 9.**
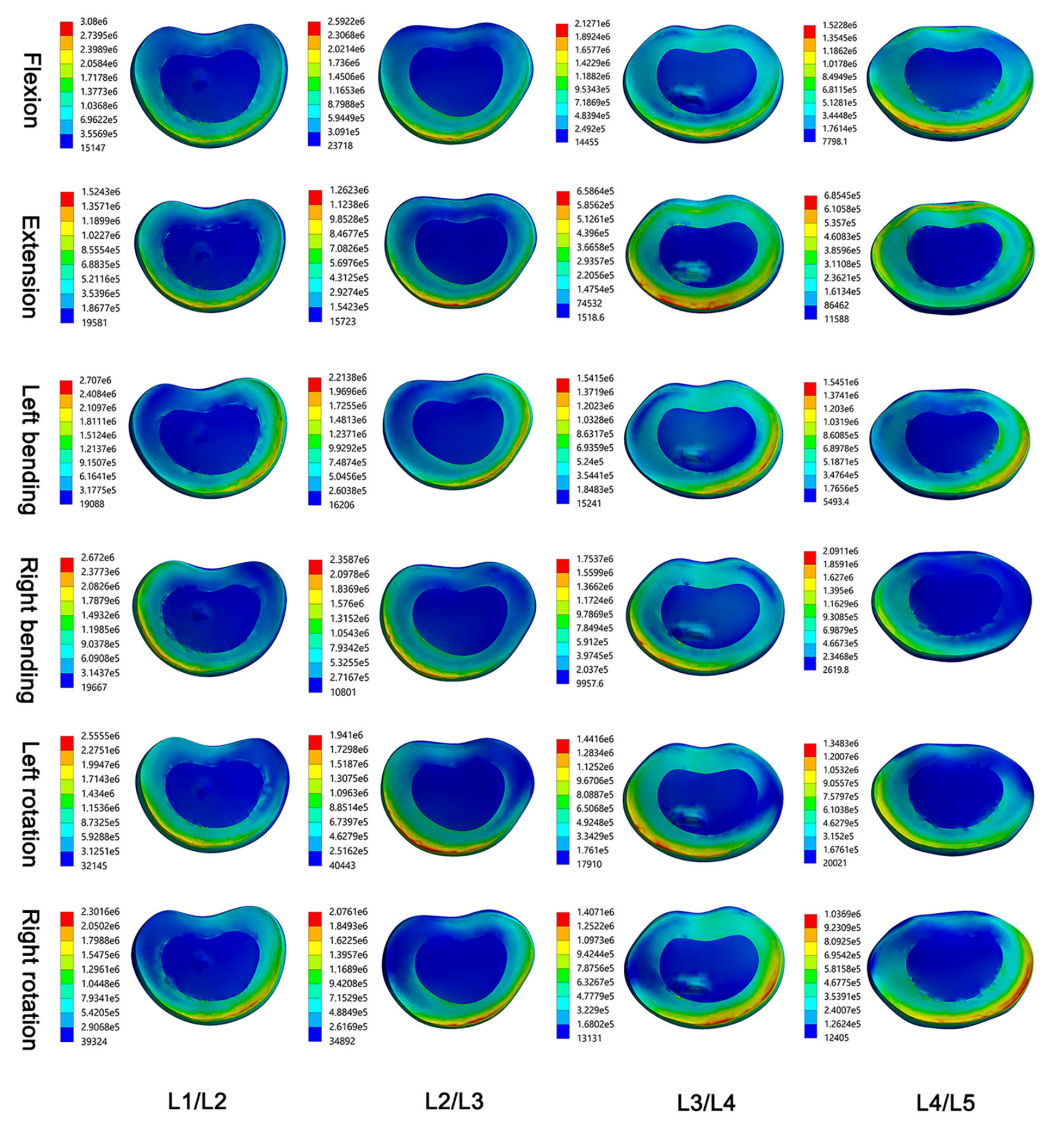
Stress distribution of intervertebral discs under flexion, extension, bending and rotation in the DTLK model. The stress values are presented in MPa. DTLK = degenerative thoracolumbar kyphosis.

**Figure 10.**
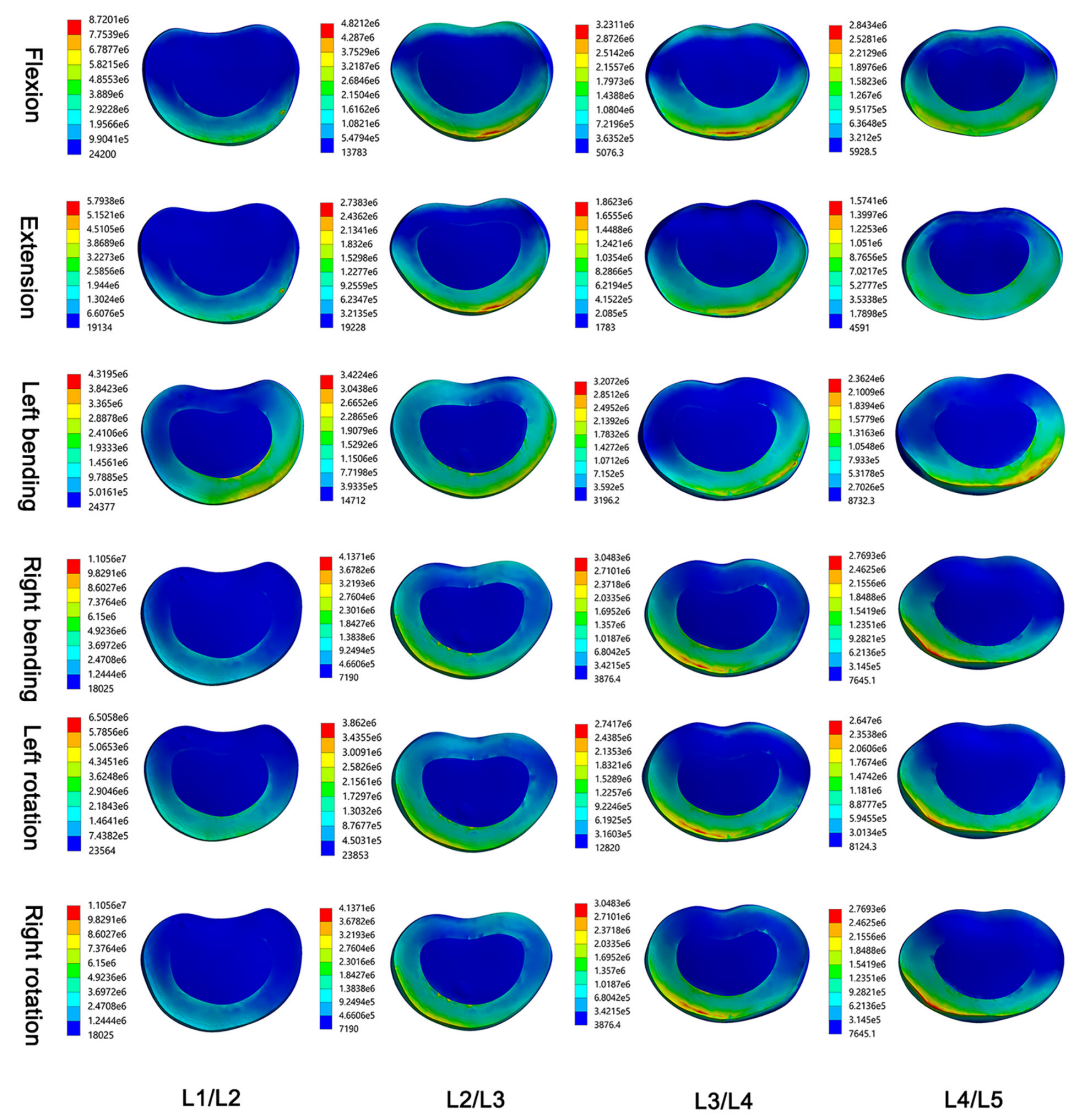
Stress distribution of intervertebral discs under flexion, extension, bending and rotation in the GK model. The stress values are presented in MPa. GK = global kyphosis.

In the normal, DTLK and GK models, the measured standing IDP values are shown in Figure 5. In the normal model, the average IDP values of T11–T12, T12–L1, L1–L2, L2–L3, L3–L4, L4–L5 and L5–S1 intervertebral discs were 0.25, 0.25, 0.24, 0.21, 0.17, 0.17 and 0.22 MPa respectively. The corresponding IDP value of DTLK model is 0.37, 0.39, 0.37, 0.33, 0.25, 0.21, 0.16 MPa. The corresponding IDP value of GK model is 0.43, 0.4, 0.52, 0.58, 0.36, 0.29, 0.25 MPa. Compared with the normal model, IDP was increased in DTLK and GK models in the standing position. It is worth noting that the intervertebral discs near the posterior convex vertex showed relatively high IDP. As shown in Figure 6, the IDP of DTLK and GK malformation models was higher than that of normal models. During flexion, extension, lateral bending and axial rotation, the average IDP values of L1–L2, L2–L3, L3–L4 and L4–L5 segments in DTLK and GK models were higher than those in normal models, and the IDP in L5–S1 segment of normal models was higher than that in DTLK. As shown in Figure 7, consistent with the simulation results of standing position, the IDP in the paravertebral region was affected by the deformity. The L2–L3 segment, located near the typical kyphotic apex, exemplified the stress elevation in deformity models. Under all simulated motions, the IDP in both the DTLK and GK models was higher than in the normal model. The values of IDP in normal models were 0.34, 0.15, 0.32, 0.32, 0.30, and 0.29 MPa during flexion, extension, left bending, right bending, left rotation and right rotation. In the DTLK model, the corresponding values are 0.45, 0.25, 0.42, 0.41, 0.39 and 0.40 MPa. In contrast, in the GK model, the values of IDP are 0.76, 0.42, 0.64, 0.64, 0.63 and 0.61 MPa. The stress distributions of the L1–L4 segmental discs under flexion, extension, lateral bending, and rotation conditions in the 3 finite element models are shown in Figures 8, 9, and 10, respectively.

### 3.5. The maximum von Mises stress in normal, DTLK, and GK models

The FEA revealed that the maximum von Mises stress in the annulus fibrosus was located in the anterior annulus during flexion and in the posterior annulus during extension. During lateral bending and axial rotation, the maximum von Mises stress was located at the lateral side of the annulus. Using the L2/L3 disc as an example, the maximum von Mises stress values in the annulus fibrosus under flexion, extension, left bending, right bending, left rotation, and right rotation were 1.96, 1.10, 1.61, 1.69, 1.31, and 1.33 MPa in the normal model, respectively. In the DTLK model, the corresponding values were 2.59, 1.26, 2.21, 2.36, 1.94, and 2.08 MPa. In the GK model, these values increased to 4.82, 2.74, 3.42, 4.14, 3.86, and 3.82 MPa.

## 4. Discussion

In the sagittal plane, the human spine is curved in an S-shape, which helps maintain balance and stability. This special anatomy allows the body’s central balance to be centered on the center of the pelvis when standing. In this ideal state, a relaxed, upright posture allows the torso to maintain the head in an appropriate position with less energy expenditure. When spinal deformity occurs, this good balance is broken. At this time, the weight of the body is distributed within the posterior articular process structure. Adult spinal deformity has been increasing in recent years with the aggravation of aging.^[23–25]^ As a subtype of Adult spinal deformity, DTLK is also closely related to IDD.^[26]^ When the intervertebral disc degenerates, the height of the intervertebral disc is reduced, resulting in an unbalanced stress distribution in each segment of the spine, and eventually leading to degenerative spinal deformity.^[27]^

We conducted a retrospective analysis of the IDD degeneration grade in T11 to S1 segments of DTLK patients. The results showed that the overall degree of disc degeneration was higher in DTLK patients than in the normal CG. In the DTLK group, there were more discs with grade Ⅳ and Ⅴ degeneration, mainly distributed in L1–L2, L2–L3, L3–L4 and L4–L5 segments. In order to further explore the mechanical mechanism of IDD in DTLK patients, we constructed a finite element model of DTLK reflecting different degrees of degeneration and a normal model. The finite element results show that the high stress region of DTLK disc is concentrated around the apex. In the normal model, the IDPs at L3–L4 (0.17 MPa) and L4–L5 (0.17 MPa) were lower than those at L1–L2 (0.24 MPa) and L2–L3 (0.21 MPa), while L5–S1 exhibited moderate stress (0.22 MPa). This distribution reflects the mechanical characteristics of the normal lumbar lordosis, with relatively lower stress in the mid-lumbar transition zone. The finite element study results are consistent with the clinical observation and provide a plausible biomechanical explanation, suggesting that the high stress concentration around the posterior convex vertex is strongly associated with and may significantly contribute to the advanced degeneration of the intervertebral disc in that region.

In our retrospective study, in patients with DTLK, grade IV and V high-level disc degeneration was mainly located in L1–L2, L2–L3, L3–L4, L4–L5 segments, especially L1–L2 and L2–L3 segments. In contrast, in the normal population, disc degeneration was mainly concentrated in the L4–L5 and L5–S1 segments. Gao A et al found that when the thoracolumbar junction is convex, the intervertebral disc is more likely to develop pathological changes, indicating that there may be a potential link between disc degeneration and abnormal mechanical stress at the convexity apex.^[28]^ Degenerative scoliosis is the most common type of spinal deformity in adults, but degenerative kyphosis is less common.^[29]^ The degeneration patterns of intervertebral discs in these 2 conditions are distinct and similar. In degenerative scoliosis, the concave surface of the scoliosis is mainly damaged by the tearing of the intervertebral disc annulus fibrosus caused by the high mechanical stress on the concave side. Similar to scoliosis, the characteristic of degenerative kyphosis is significant degeneration of the anterior column annulus fibrosus, which is the result of anterior shear force. The apical vertebra of kyphosis or scoliosis deformity serves as a transition zone between the proximal and distal segments of the spine, with high mechanical load, which is prone to stress concentration, which may accelerate disc degeneration.^[30]^ Zhang Q et al found that in degenerative scoliosis, the degeneration of the vertebral apex was more severe than other segments.^[31]^ Yeung KH et al observed that in adults with scoliosis, the nucleus pulposus of the intervertebral disc at the apex of the scoliosis is compressed due to the imbalance of stress between the concave and convex sides, which eventually leads to disc degeneration.^[32]^ Although direct literature on thoracolumbar kyphotic disc degeneration is limited, related conditions such as Scheuermann kyphosis exhibit similar degenerative patterns at the apex segments, supporting our biomechanical observations. The mechanical stress on the anterior column of the DTLK and GK discs is similar to the stress on the concave surface of the scoliosis deformity. When degenerative kyphosis occurs, the pressure on the anterior column exceeds the stress on the posterior column.

In order to explore the mechanical mechanism of spinal deformity (IDD), we constructed 3 models: normal model, thoracolumbar kyphosis model and GK model. The spinal finite element modeling method has been fully validated, and this technique has wide application value in the study of spinal biomechanics.^[33]^ FEA can be used to evaluate the mechanical changes in degenerative kyphosis in detail. Although the intervertebral disc stress values calculated by FEA may be slightly different from the actual physiological values, the overall stress distribution pattern can effectively reflect and explain the biomechanical mechanisms involved in disease progression.^[34]^

In the finite element study, we observed that the stress in the distal spine of the DTLK model was lower than that in the thoracolumbar region. The occurrence of this phenomenon can be explained by the posterior pelvic tilt and sacral leveling in kyphotic deformity. Due to the posterior pelvic tilt and sacral leveling, the L5–S1 segment in the DTLK and GK models becomes more horizontal, redirecting gravitational load toward the thoracolumbar segment apex rather than the lumbosacral junction. Additionally, the loss of lumbar lordosis shifts the sagittal alignment axis anteriorly, potentially reducing disc stress at the L5–S1 level. Therefore, distal vertebral segments and intervertebral discs are less affected by spinal deformities. Li J et al reported that mechanical stress is most likely to concentrate in the top region of the spine under gravitational load, especially on the concave side of the apical vertebra.^[35]^ Similar conclusions were found in our finite element model, where the overall trend of intervertebral disc stress was significantly higher in the DTLK model and the GK model than in the normal model. However, the normal model disc stress in L5–S1 segment was greater than that of DTLK model. The DTLK model and GK model showed higher intervertebral disc stress than normal models in L1–2, L2–3, L3–4 and L4–5 segments. Taking L1–2 segment as an example, compared with the normal model, the IDP and maximum von Mises stress at L1–L2 level in GK model were significantly increased. In a study of the effect of sagittal morphology on disc stress, Matsumoto K et al found that disc stress also increases with increasing sagittal vertical axis.^[9]^ Consistent with their results, our results showed that the disc pressure of GK model was greater than that of DTLK. This suggests that IDP increases with the severity of thoracolumbar kyphosis. Shen Y et al suggested that the increase in IDP may be related to increased shear forces caused by convex deformity.^[36]^ This observation may provide some ideas for explaining the effects of different vertices and the degree of sagittal imbalance on disc degeneration.

Our mechanical analysis of the 3 spinal models also found that as the degree of kyphosis increased, so did the stress in front of the intervertebral disc. In the normal model, the stress is basically uniformly distributed in front and back, but in the DTLK model and GK model, the high stress distribution area of each intervertebral disc is basically located in the front of the intervertebral disc. When degenerative kyphosis occurs, the pressure load on the anterior column of the spine increases significantly, especially in the intervertebral disc at the apex segment of kyphosis. At this time, abnormal shear forces increase the pressure and friction of the articular joint, resulting in cartilage wear and narrowing of the joint space. Prolonged exposure to these shear forces can also lead to ligament laxity and muscle strain around the articular joint, which accelerates articular degeneration. Ultimately impairing the function of the posterior spinal tension band. The combination of increased compressive load on the anterior column and loss of posterior tension band support changes the pressure distribution of the intervertebral disc. These modified mechanical stresses, combined with abnormal shear forces, compress the nucleus pulposus, accelerate the loss of water in the nucleus pulposus, and induce damage to the annulus fibrosus.

## 5. Limitations

Our study also has some limitations. First, osteoporosis may affect IDD. Although our patient cohort was older, osteoporosis was not accounted for in our analysis. Second, the finite element model was constructed without considering the role of paravertebral muscles. In addition, we employed a fixed-point load rather than a follower load to ensure comparability across models, which may not fully capture the dynamic anterior load distribution during spinal bending. Future research should incorporate the characteristics of osteoporosis and the influence of paravertebral muscles on intervertebral disc stress to achieve a more comprehensive understanding of the mechanical mechanisms underlying IDD.^[37]^

## 6. Conclusions

DTLK exhibits a distinctive pattern of IDD predominantly concentrated at the L1–L4 segments, contrasting sharply with the L4–S1 predominance commonly observed in normal aging. FEA demonstrates that this unique distribution is biomechanically associated with increased intradiscal pressure and anterior annulus fibrosus stress concentration at the kyphotic apex. These findings provide a biomechanical basis for understanding the pathogenesis of DTLK.

## Author contributions

**Conceptualization:** Xilong Cui.

**Data curation:** Wanmei Yang, Ao Ding.

**Funding acquisition:** Kangkang Wang.

**Investigation:** Jian Wang.

**Methodology:** Ji Li.

**Project administration:** Wei Zhang.

**Writing – original draft:** Ruifeng Xun.

**Writing – review & editing:** Haiyang Yu.
